# Real-Time Observation of Polymer Fluctuations During Phase Transition Using Transmission Electron Microscope

**DOI:** 10.3390/polym17030292

**Published:** 2025-01-23

**Authors:** Takaaki Shiina, Tatsunari Ohkubo, Keegan McGehee, Rena Inamasu, Tatsuya Arai, Daisuke Sasaki, Yuji C. Sasaki, Kazuhiro Mio

**Affiliations:** 1 AIST-UTokyo Advanced Operando-Measurement Technology Open Innovation Laboratory (OPERANDO-OIL), National Institute of Advanced Industrial Science and Technology (AIST), 6-2-3 Kashiwanoha, Chiba 277-0882, Japan; shiina-takaaki@aist.go.jp (T.S.); ookubo.t@aist.go.jp (T.O.); ycsasaki@edu.k.u-tokyo.ac.jp (Y.C.S.); 2 Graduate School of Medical Life Science, Yokohama City University, 1-7-29 Suehiro-cho, Tsurumi-Ku, Yokohama 230-0045, Japan; 3 Graduate School of Frontier Sciences, The University of Tokyo, 5-1-5 Kashiwanoha, Chiba 277-8561, Japant.arai@elms.hokudai.ac.jp (T.A.);; 4 Department of Advanced Transdisciplinary Sciences, Faculty of Advanced Life Science, Hokkaido University, Kita 10, Nishi 8 Kita-ku, Sapporo 060-0810, Japan

**Keywords:** membrane dynamics, transmission electron microscope, nano-particle tracking, operando measurement, in situ thermal control

## Abstract

Measuring molecular dynamics improves understanding of the structure–function relationships of materials. In this study, we present a novel technique for observing material dynamics using transmission electron microscopy (TEM), in which the gold nanoparticles are employed as motion probes for tracing the polymer dynamics in real space. A thin layer of polymer materials was generated on the 2 μm diameter holes of Quantifoil grids, and gold nanoparticles were dispersed on the membrane surface. By tracking the movement of gold nanoparticles from a series of TEM images taken under continuous temperature control, we obtained mean squared displacement (MSD) curves. The dynamics of poly{2-(perfluorooctyl)ethyl acrylate} (PC_8_FA) and poly(stearyl acrylate) (PSA) were analyzed. In the temperature-dependent analysis of the MSD, sharp peaks were observed for both PC_8_FA and PSA at positions corresponding to their melting and crystallization temperatures. These results demonstrate the capability of TEM to provide valuable insights into the dynamics of polymer materials, highlighting its potential for widespread application in materials sciences.

## 1. Introduction

Measurement of molecular dynamics improves understanding of the structure–function relationships of materials including polymers and proteins [1,2]. Various techniques such as optical microscopy, high-speed atomic force microscopy, X-ray free electron laser, and electron microscopy have been used for this purpose [3,4,5,6,7,8,9,10,11,12,13,14,15,16]. Recent advances in electron microscopy have enabled the observation of chemical and physical processes directly in situ with high spatial and temporal resolution [17,18,19]. Using micro-sample installation devices such as MEMS chips and environmental cells composed of SiNx, graphene chambers, or carbon sandwiches, extensive research is being conducted. Examples include the study of chemical reactions, crystal formation, and other phenomena in liquid or gas environments, under various physical stimuli such as electric, thermal, and mechanical control [20,21,22,23,24,25]. Additionally, distinctive applications of single-particle analysis using cryo-electron microscopy, such as time-resolved studies of molecular conformation changes to elucidate protein dynamics, have been actively pursued [26,27,28,29]. Furthermore, high-resolution electron microscopy techniques have visualized the movements of molecules, including perfluoroalkyl chains and antibiotics, at the atomic level [30,31,32]. Ultrafast electron microscopy is another state-of-the-art technique capable of examining objects at femtosecond and angstrom scales [33,34]. These technical advancements underscore that transmission electron microscopy (TEM) is one of the most powerful tools for molecular dynamics research, with growing demand expected across many fields of modern science and engineering.

Our research group has developed X-ray-based time-resolved motion analysis techniques called diffracted X-ray tracking (DXT) and diffracted X-ray blinking (DXB) [35,36]. DXT tracks the movement of X-ray diffraction patterns generated from the materials, providing molecular-level dynamic information, while DXB acquires dynamic data from the fluctuation in the blinking intensity of the diffraction patterns. These methods enable us to observe dynamics of a wide variety of materials and biomolecules with high spatiotemporal resolution (on the order of nanoseconds and picometers) in a highly adjustable experimental environment [1,35,36,37,38,39,40,41,42,43,44,45,46,47]. The principles of DXT can also be applied to diffraction from electron beams in SEM, a technique called diffracted electron tracking (DET) [48,49,50].

In this study, we developed a novel molecular dynamics observation technique using TEM by applying the particle tracking method, originally established in the DXT and DXB techniques. Gold nanoparticles were used as probes to monitor motion. The 5 nm diameter gold nanoparticles were dispersed on the sample surface, and their movement was directly recorded using a widely available conventional TEM equipped with a CCD camera. For the experimental design, we applied repetitive heating and cooling cycles as stimuli during observation to investigate the in situ dynamics of the polymer samples.

For our initial study, we observed the motion of the fluorinated acrylate polymer PC_8_FA (poly{2-(perfluorooctyl)ethyl acrylate}), which is known for its high water and oil repellency, non-adhesive properties, low friction coefficients, and antifouling behaviors [45,46,51,52,53,54,55,56,57,58,59,60]. For comparison, we also analyzed the motion of poly(stearyl acrylate) (PSA), a water-repellent material [54].

## 2. Experimental Methods

### 2.1. Preparation of TEM Samples

PC_8_FA powder was synthesized by free radical polymerization of perfluorooctylethyl acrylate (C_8_FA) using a method reported previously [46]. The number average molecular weight (Mn), weight average molecular weight (Mw), and molecular weight dispersity (MWD) of the polymer were measured by GPC as 28,900 g/mol, 74,500 g/mol, and 2.58, respectively. The polymer powder dissolved in the solvent AK-225 (a mixed solvent of 1,1-dichloro-2,2,3,3,3-pentafluoropropane and 1,3-dichloro-1,1,2,2,3-pentafluoropropane) to prepare a 0.2 wt % solution. Aqueous gold nanoparticles with a 5 nm diameter (BBI Solutions, Crumlin, UK) were dispersed using a Branson M1800-J Ultrasonic Bath (Branson Ultrasonics, Brookfield, CT, USA) for 10 min. A 2 μL aliquot of the PC_8_FA solution was placed onto a Quantifoil Cu #200 R2/2 grid (Quantifoil Micro Tools GmbH, Großlöbichau, Germany). Before the solvent had fully evaporated, 4 μL of the gold nanoparticle solution was quickly added within 2~3 s. Excess gold nanoparticle solution was removed using blotting paper. The sample was air-dried for several minutes to evaporate the tiny residue of water.

PSA was prepared by free radical polymerization. Mn, Mw, and MWD of the polymer were measured by GPC as 19,500 g/mol, 28,700 g/mol, and 1.48, respectively. The polymer powder was dissolved in chloroform (FUJIFILM Wako Pure Chemical Corporation, Osaka, Japan) to make a 0.5 wt% solution. The gold nanoparticle solution was dispersed using an ultrasonic bath for 10 min. A 2 μL aliquot of the PSA solution was placed onto a Quantifoil grid. Before the solvent had fully evaporated, 4 μL of the gold nanoparticle solution was added. Excess gold nanoparticle solution was removed using blotting paper. The sample was air-dried for several minutes to evaporate the tiny residue of water.

### 2.2. TEM Observation

TEM observation was conducted using a JEM 1230 (JEOL Ltd., Tokyo, Japan) at an accelerating voltage of 120 kV with a magnification of 40,000×. The current density during observation ranged from 3 to 12 pA/cm^2^. Images were acquired using a CCD camera (FastScan-F114, TVIPS GmbH, Gauting, Germany) under the control of EM-MENU (TVIPS GmbH). The FastScan-F114 has 1024 × 1024 pixels, with each pixel sized 14 × 14 μm^2^. Real-time TEM videos were recorded using OBS Studio (OBS Project) by capturing the EM-MENU window at 12 fps.

Temperature control of the specimens was conducted using a Peltier heating/cooling sample holder (KITANO SEIKI Co., Ltd., Tokyo, Japan). The TEM specimens were initially cooled from room temperature to 0 °C, then heated from 0 °C to 98 °C under PID control, with the temperature change rate maintained at 0.1 °C/s or lower. After reaching 98 °C, the specimens were cooled back to 0 °C. These heating/cooling cycles were repeated.

### 2.3. Mean Squared Displacement and Probability Density Analysis

Video data were converted to continuous image data using FFmpeg (ffmpeg.org accessed on 16 December 2024). Each image was processed using ImageJ (Rasband, W.S., ImageJ, U.S. National Institutes of Health, Bethesda, ML, USA, https://imagej.net/ij/, 1997–2018, accessed on 16 December 2024). Background correction, black/white inversion, and brightness and contrast adjustments were applied to the data for optimal particle identification.

Particle identification and mean square displacement (MSD) calculation were performed using the TrackPy program (v0.3.2, https://doi.org/10.5281/zenodo.60550) for the processed continuous images. The MSD curves were analyzed using custom software created in IGOR Pro version 9 (Wavemetrics, Lake Oswego, OR, USA). The MSD value was calculated from the size of gold nanoparticles in the processed images by ImageJ. The resulting pixel size was calculated as 0.392 nm. To calculate MSD, 135 gold nanoparticles were analyzed for PC_8_FA and 35 gold nanoparticles for PSA.

Probability density (PD) was calculated at Δt = 8 s, plotted and curve-fitted using IGOR Pro. Each histogram was fitted with Gaussian curves, and boxplots were generated from the PD data. For each experiment, we recorded 121 frames at a frame rate of 12 frames per second, corresponding to 10 s of data. For each nanoparticle, we identified 25 displacements corresponding to Δt = 8 s. In total, we obtained approximately 3000 displacements for PC_8_FA and 700 displacements for PSA. Near the transition temperatures, nanoparticle motion increased, causing some nanoparticles to move out of the field of view or reducing contrast, and the ‘n’ values near T_m_ and T_c_ were lower than those in other temperature regions.

### 2.4. Differential Scanning Calorimetry (DSC)

DSC measurements were performed using a DSC 3 (Mettler Toledo International Inc., Greifensee, Switzerland). Approximately 3.5 mg of sample powder was sealed in 25 μL aluminum pans with lids. Using N_2_ gas as a protective gas, heating and cooling cycles were conducted between 0 °C and 100 °C with a rate of 5 °C/min. The cycle was repeated several times.

## 3. Results

**Figure 1:**
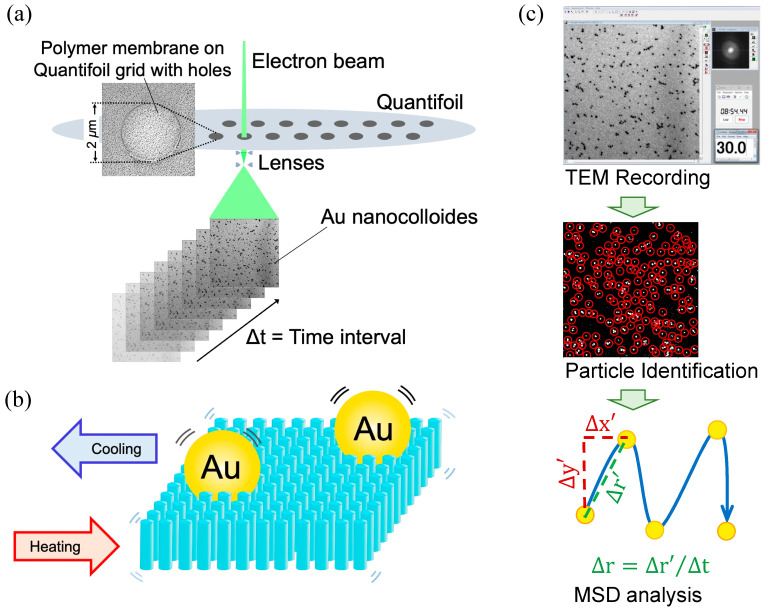
Experimental procedure and analysis: (**a**) Schematic illustration of TEM-based direct observation of thermally induced polymer fluctuations. (**b**) Images of gold nanoparticles embedded on the polymer surface. (**c**) Flowchart of the data analysis process.

To observe thermal-induced polymer fluctuation using electron microscopy, 5 nm diameter gold nanoparticles were used (Figure 1a,b). A thin layer of polymer films was generated on a Quantifoil Cu #200 R2/2 grid, which contains 2 μm diameter holes supported by a carbon frame, and the gold nanoparticles were dispersed on the surface. The grid was loaded onto the temperature-controllable Peltier sample holder, and the motion of the gold nanoparticles was recorded during continuous heating and cooling cycles using a JEM1230 electron microscope equipped with a TVIPS FastScan-F114 CCD camera. From the video data, the MSD and PD of the gold nanoparticle movement were analyzed (Figure 1c, Supplementary Figure S1). Due to the limitation of video capture, data were recorded with a frame rate of 12 fps.

### 3.1. Motion Analysis of PC_8_FA

**Figure 2:**
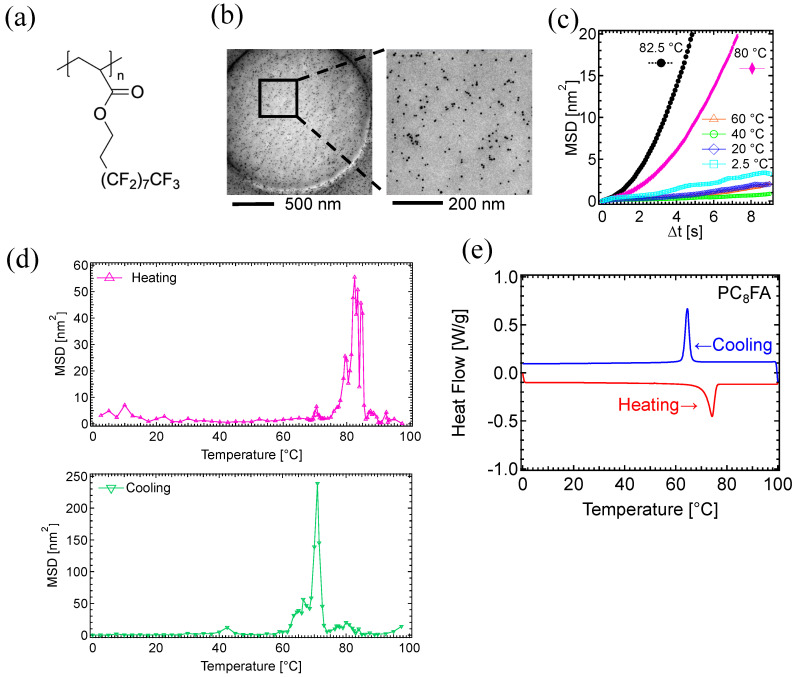
TEM-based motion analysis of PC_8_FA in heating/cooling cycles: (**a**) Chemical structure of PC_8_FA. (**b**) TEM image of gold nanoparticles on the PC_8_FA membrane. (**c**) MSD curves of PC_8_FA in heating process. (**d**) MSD of (upper) the heating processes from 0 to 98 °C, and (lower) the cooling processes from 98 to 0 °C. Data were obtained from the third cycle of the continuous heating and cooling processes. MSDs at specific time intervals of 8 s (Δt = 8 s) were analyzed. (**e**) DSC results of PC_8_FA.

The motion of PC_8_FA (Figure 2a) was analyzed. A TEM image of the 2 μm hole reveals well-dispersed gold nanoparticles on the PC_8_FA polymer film, which was prepared on a Quantifoil grid (Figure 2b).

Movie data were recorded under heating and cooling condition between 0–98 °C using the JEM 1230 electron microscope with a beam current of 3.0 [e/Å^2^s] at the specimen level. The heating rate of the equipment was 3 °C to 5 °C/min, while the cooling rate ranged from −2 °C to −6 °C/min (Supplementary Figure S2). The temperature change rates below 10 °C (0–10 °C) and over 80 °C (80–98 °C) were lower than those observed between 10 °C and 80 °C. As the crystallographic quality of PC_8_FA improves through annealing after chemical or physical damage by self-healing [51,59,60], the heating and cooling cycles were repeated several times (Supplementary Figure S3) and the dynamics of the third heating and cooling cycles were presented.

MSD curves of PC_8_FA at 20 °C intervals demonstrated a significant increase in the curve angle at 80 °C (Figure 2c), which is close to the reported melting temperature of PC_8_FA ranging 70–75 °C [46,51,56,57,58]. In the figure, the MSD curve of 82.5 °C was also depicted.

MSD values against temperature highlight the temperature-dependent dynamics change of polymers (Figure 2d). Preliminary experiments comparing the MSD values at one-second intervals from Δt = 1 s to Δt = 9 s suggested that the MSD value at Δt = 8 s is the most effective for comparing temperature-dependent differences and aligns well with previous research on the temperature- and time-dependent oil repellency of PC_8_FA [56]. Therefore, subsequent data focused on the MSD values at Δt = 8 s.

The MSD of the heating process, measured at 0.5 °C intervals, showed a significant increase in MSD between 75 °C and 85 °C (Figure 2d, upper). Considering the reported melting temperature of PC_8_FA (70–75 °C), this large MSD peak reflects molecular motion associated with structural remodeling in melting. The MSD peak consisted of at least three distinct sub-peaks at 79.5 °C, 82.5 °C, and 84.5 °C, with the highest peak at 82.5 °C.

The presence of multiple peaks near the melting temperature may be explained by the heterogeneity of the PC_8_FA sample analyzed in this study. The sample consists of fluoroalkyl side chains that are prone to crystallization and a polycarbonate main chain, along with a broad molecular weight distribution, as indicated by the high molecular weight dispersity (MWD ≈ 2.58).

We then analyzed the motion dynamics of PC_8_FA during the cooling process. As the sample temperature decreased from 98 °C to 0 °C, the motion of the gold nanoparticles was recorded. During the cooling cycles, a sharp peak in the MSD was observed at 71 °C, with a slight shoulder between 60.0 °C and 67.5 °C (Figure 2d, lower). The temperature at the MSD peak position was 11.5 °C lower in the cooling cycle compared to that of the heating cycle. During crystallization upon cooling, a temporary overcooling state may occur [61,62,63,64]. At the crystallization point, multiple crystallizing process may take place almost simultaneously. A rearrangement of the crystal structure could be occurring at the shoulder peaks between 60.0 °C and 67.5 °C.

MSD peaks obtained from the TEM analysis were compared with the dynamic quantities from the calorimetric data. The DSC measurements were performed on a powder PC_8_FA sample, with heating and cooling cycles applied between 0 °C and 100 °C at a rate of 5 °C/min. The DSC analysis demonstrated that the melting temperature (T_DSC, melt_) and crystallization temperature (T_DSC, crystal_) for PC_8_FA were 74 °C and 65 °C, respectively (Figure 2e).

In the heating process, a significant MSD peak was observed around 82.5 °C by TEM (Figure 2d), which is slightly higher but closely matches the reported melting point (T_m_ ≈ 70–75 °C), as well as our DSC analysis (74 °C). In the cooling process, the MSD peak observed by TEM at 71 °C also closely matches, but is slightly higher than the DSC peak at 65 °C. The temperature gap between the melting and the crystallization points (i.e., T_m_ − T_c_) was 11.5 °C for TEM and 9 °C for DSC.

**Figure 3:**
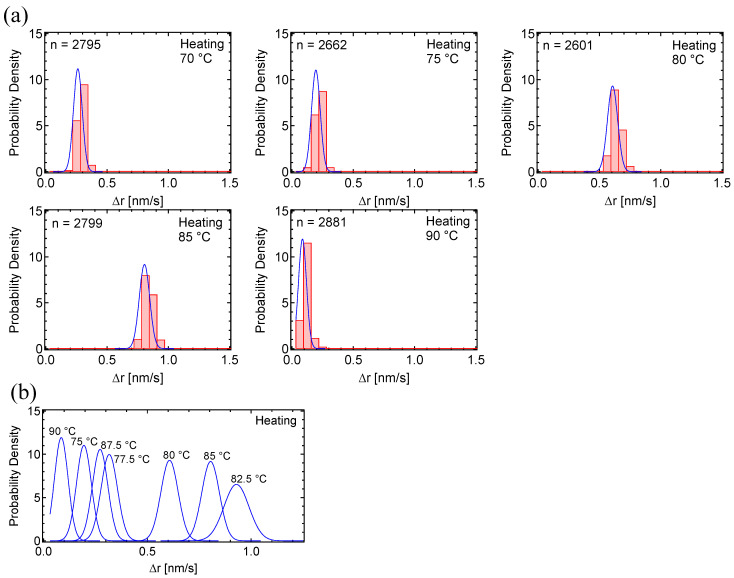
Probability density histograms of PC_8_FA in heating cycles: (**a**) PD histograms of PC_8_FA under the heating process, taken at 5 °C intervals between 75–90 °C. (**b**) Overlay of fitting curves for the PC_8_FA heating process. In the figures, ‘n’ represents the total number of displacements.

We analyzed the PD histograms of the motion component Δr’ = (Δx’^2^ + Δy’^2^)^1/2^, where x’ is the horizontal and y’ is the vertical movement of each gold nanoparticle in the TEM images. Motion histograms during the heating process, taken at 5 °C intervals between 70–90 °C (Figure 3a), and the overlay of the fitting curves (Figure 3b) demonstrate that the motion increased between 77.5–87.5 °C. The motion histograms at this temperature range were well-fitted by single curves. The maximum motion dynamics was calculated as 0.92 nm/s at 82.5 °C.

**Figure 4:**
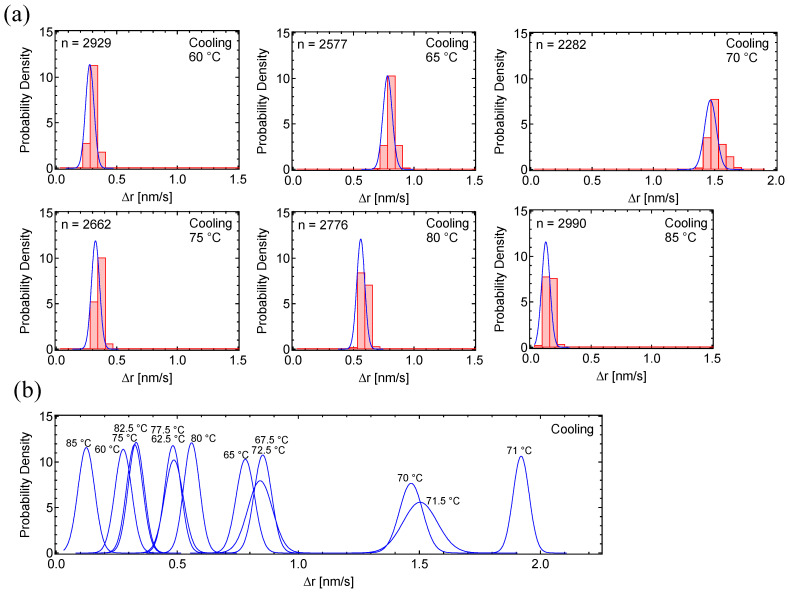
Probability density histograms of PC_8_FA in cooling cycles: (**a**) PD histograms of PC_8_FA under the cooling process, taken at 5 °C intervals between 60–85 °C. (**b**) Overlay of fitting curves for the PC_8_FA cooling process. In the figures, ‘n’ represents the total number of displacements.

Motion histograms during the cooling process, taken at 5 °C intervals from 85 °C to 60 °C (Figure 4a), and the overlay of their curve fittings (Figure 4b), show that the motion increased between 67.5 °C and 72.5 °C. The motion histograms in this temperature range were also well-fitted by single curves. The maximum motion dynamics of 1.9 nm/s at 71 °C (Figure 4b) was almost double the 0.92 nm/s observed during the heating process (Figure 3b). Since the crystallization process occurs rapidly within a narrow temperature range, it is reasonable to observe more rapid peak dynamics in the cooling process, which may be significantly high.

We carefully selected well-dispersed areas for TEM recording. However, after closer inspection, we did find some instances of 2–3 particle clusters being tracked together. To evaluate the size effect of gold nanoparticles on MSD, we categorized the nanoparticles into two size groups: those with diameters of 4–6 nm (representing individual gold nanoparticles) and those with diameters of 9–11 nm (representing clusters of 2–3 gold nanoparticles, which were tracked as single entities in the MSD calculations). From each size group, we analyzed 10 sets of particles to obtain MSD values.

Our results showed that the nanoparticle size had minimal impact on the MSD measurements. Specifically, there were no significant differences in the temperatures at which the MSD peaks occurred (T_m_ and T_c_) across the two size groups (Supplementary Figure S4).

### 3.2. Motion Analysis of PSA

**Figure 5:**
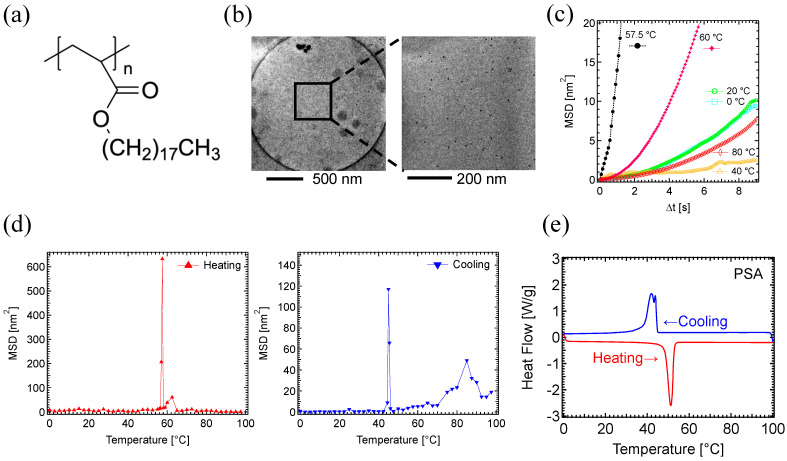
TEM-based motion analysis of PSA in heating/cooling cycle: (**a**) Chemical structure of PSA. (**b**) TEM image of gold nanoparticles on the PSA membrane. (**c**) MSD curves of PSA in heating process. (**d**) MSD of (**left**) heating process from 0 to 98 °C, and (**right**) cooling process from 98 to 0 °C. MSDs at specific time intervals of 8 s (Δt = 8 s) were analyzed. (**e**) DSC results of PSA.

To confirm whether the change in MSD values correlates with the state transition of the polymer materials, we next analyzed the dynamics of a PSA sample (Figure 5a), which has a reported T_m_ of 50 °C [54], approximately 25 °C lower than that of PC_8_FA (T_m_ ≈ 70–75 °C).

A TEM image of the PSA sample also revealed well-dispersed gold nanoparticles on the polymer membrane surface (Figure 5b). The MSD curves of PSA during heating taken at 20 °C intervals showed that the slope at 60 °C was much higher than at other temperature conditions (Figure 5c). In the figure, the steepest MSD curve of 57.5 °C is also depicted. The MSD values as a function of temperature demonstrated steeper and narrower peaks compared to those of PC_8_FA (Figure 2d). TEM analysis showed the T_m_ of PSA in the heating process was 57.5 °C, which is 25 °C lower than that of PC_8_FA (T_m_ ≈ 82.5) (Figure 5d, left). The T_c_ of PSA in cooling process was 45 °C, which is 26 °C lower than that of PC_8_FA (T_c_ ≈ 71 °C) (Figure 5d, right).

We again analyzed PSA using DSC calorimetry (Figure 5e). The DSC value for PSA was T_DSC, melt_ ≈ 52 °C in the heating process (22 °C lower than that of PC_8_FA) and T_DSC, crystal_ ≈ 45 °C in the cooling process (20 °C lower than that of PC_8_FA) (Figure 5e). The T_m_ and T_c_ values obtained from TEM method aligned well with the temperature differences reported for PC_8_FA and PSA, which was further supported by the DSC analysis.

**Figure 6:**
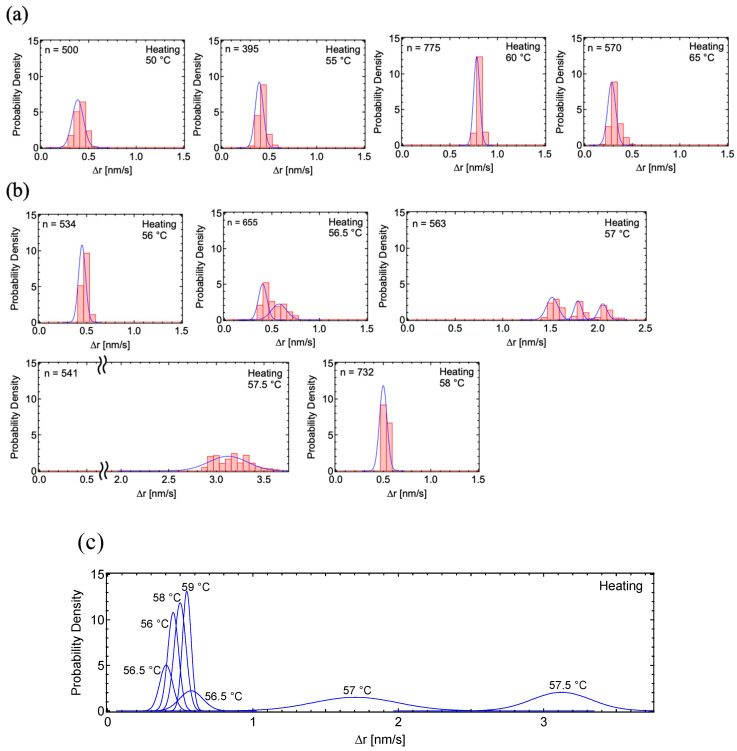
Probability density histograms of PSA in heating cycles: (**a**) PD histograms of PSA under the heating process, taken at 5 °C intervals between 50–65 °C. (**b**) Detailed PD histograms of PSA under the heating process, taken at 0.5 °C intervals between 56–58 °C. (**c**) Overlay of fitting curves for the PSA heating process. In the figures, ‘n’ represents the total number of displacements.

Motion histograms during the heating process, taken at 5 °C intervals between 50–65 °C, demonstrated that the motion dynamics were enhanced between 55 °C and 65 °C (Figure 6a). To investigate the dynamics in more detail, we analyzed the PD histograms at 0.5 °C intervals between 56 °C and 58 °C (Figure 6b). In the transition state, the PD histograms were divided into several groups with different motion speeds, and these were fitted by two Gaussian curves at 56.5 °C and three Gaussian curves at 57 °C. The maximum motion dynamics of 3.1 nm/s at 57.5 °C for PSA (Figure 6c) was significantly higher than the 0.92 nm/s observed during the heating process for PC_8_FA (Figure 3b).

**Figure 7:**
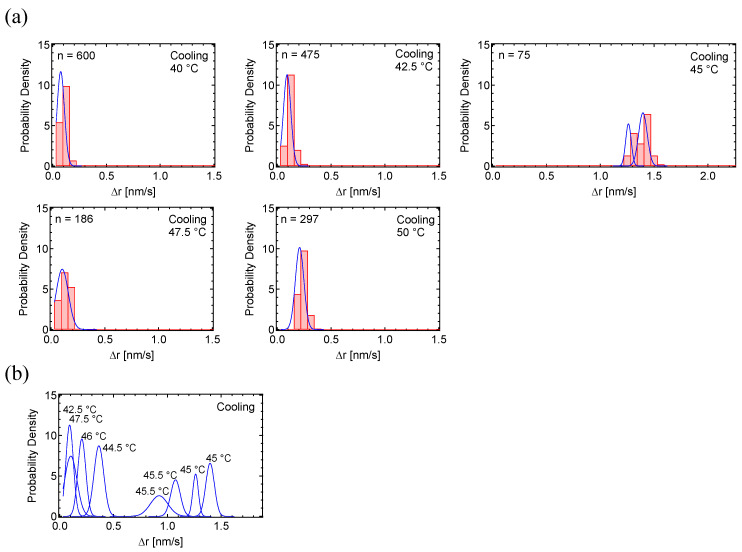
Probability density histograms of PSA in cooling cycles: (**a**) PD histograms of PSA under the cooling process, taken at 2.5 °C intervals between 40–50 °C. (**b**) Overlay of fitting curves for the PSA cooling process. In the figures, ‘n’ represents the total number of displacements.

Motion histograms during the cooling process, taken at 2.5 °C intervals from 50 °C to 40 °C (Figure 7a), and the overlay of their fitting curves (Figure 7b), show that the motion increased between 42.5 °C and 47.5 °C. The motion in the transition state at 45 °C was fit by two Gaussian curves, while the PD of other temperatures were well-fitted by single curves. The maximum motion dynamics of 1.4 nm/s at 45 °C (Figure 7b) was lower than the 3.1 nm/s observed during the heating process (Figure 6c). A broad MSD peak was observed for PSA between 70 °C and 90 °C in the cooling process (Figure 5d, right). This may partly explain the lower MSD value of PSA at the crystallization point.

**Figure 8:**
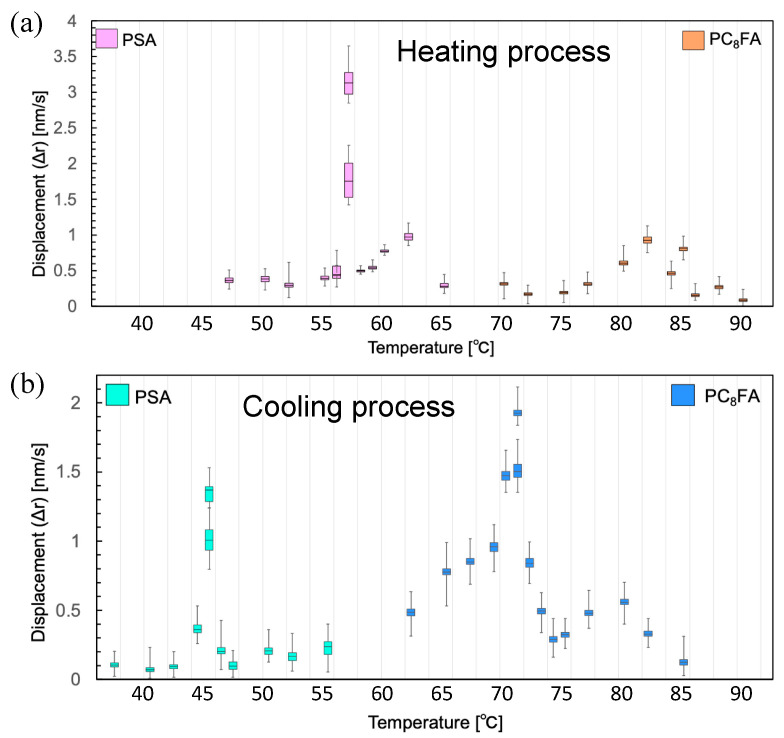
Boxplot analysis of PC_8_FA and PSA in heating/cooling cycles. Boxplot analysis of PC_8_FA and PSA in (**a**) the heating process and (**b**) the cooling process. Box plots represent the minimum, 25th percentile, 50th percentile, 75th percentile, and maximum.

The box plots clearly demonstrate the temperature-dependent motion of PC_8_FA and PSA at both the melting and crystallization points (Figure 8). The box plots for PC_8_FA during the heating process displayed a widely dispersed distribution pattern, with the peak MSD value significantly lower than that of PSA (Figure 8a). However, no significant difference was observed in the peak MSD values between PC_8_FA and PSA during the cooling process (Figure 8b). This suggests that the PC_8_FA sample exhibits more intramolecular or intermolecular heterogeneity than the PSA sample in this study.

## 4. Discussion

In this study, we introduce a novel approach for analyzing polymer dynamics using TEM. We successfully captured thermally induced polymer fluctuations by employing 5 nm gold nanoparticles as motion monitors. The MSD peaks obtained from the TEM measurements corresponded well with the calorimetric data obtained from DSC.

Distinct MSD behaviors were observed during phase transitions, such as melting in heating cycles and crystallization in cooling cycles. These behaviors diminished once the phase transition was complete. The difference between T_m_ and T_c_ arose because T_m_ is thermodynamic, while T_c_ is kinetic. Crystallization requires chain alignment and nucleation, which occur at lower temperatures than melting, causing supercooling [61,62,63,64]. This trend was also seen in our DSC measurements as a temperature difference between cooling and heating peaks. The repeated heating/cooling cycle of PC_8_FA was shown to induce an annealing effect, improving the crystallographic orientation and regularity of the R_f_ side chain [46,51], which could be reflected in changes to the MSD peak behavior. These phenomena were consistently reproduced in our TEM measurements, supporting the theoretical reliability of this method.

The diffusion coefficient D of the PC_8_FA membrane was calculated at 20 °C using the equation D = MSD^2^/2dΔt, where d is the dimension of motion, and Δt is time intervals. In these experiments, d = 2 and Δt = 8 s. The resulting value 1.83 × 10^−1^ nm^2^/s was 5.55 × 10^4^ times larger than the rotational diffusion coefficient 3.29 pm^2^/s obtained by DXB measurement [45]. The difference may come from the fact that the DXB measured at a smaller timescale (~1 s) and for a smaller object size (~0.5 nm), primarily focusing on the motion analysis of the fluoroalkyl side chain.

Electron damage to samples must be carefully considered. To minimize damage, many EM experiments such as direct observation of polymer structure and cryo-EM single-particle analysis were conducted at very low electron doses [2,14,26,27,28,29,65]. The electron dose in this study was reduced to a level that allows particle tracking in video images (3–12 pA/cm^2^), but the total amount of electron irradiation throughout the experiment remained relatively high. Although there is unequivocal evidence of irradiation-induced damage occurring in this experiment, considering reproducible data among the repeated measurement cycles, it does not seem to be a critical issue in this study. We used a conventional TEM and CCD detector here, but in future work, the use of high-sensitive cameras could reduce electron dose and enable more detailed analysis.

Compared to the PSA, the main MSD peak of PC_8_FA during the heating process was broad and contained at least three sub-peaks. This suggests that the PC_8_FA sample exhibits greater intramolecular or intermolecular heterogeneity compared to the PSA sample in this study. The characteristic melting behavior of PC_8_FA is also inferred, as it transitions into an amorphous state at temperatures between 74 °C to 84 °C, losing its lamellar structure and side chain alignment [57,58]. The existence of several sub-peaks in the MSD main peak may also reflect the gradual transformation and loss of the crystal structure during melting.

Our previous GI-DXB study on the dynamics of PC_8_FA demonstrated that the decay constant distribution for the R_f_ side chain is broad, while that for the lamellar structure is narrow [46]. This corresponds with the PD distribution observed in this TEM analysis. When the PD distribution is narrow, it likely reflects the movement of the lamellar structure. At the transition point, the shift of Δr to larger values and the broadening of the distribution may result from the increased movement of some side chains, while the movement of other side chains, the main chain, or parts of the lamellar structure slows down. Polymer dynamics may involve multiple layers of motion.

Based on these observations, we propose that our results primarily reflect a shift in imaging related to molecular dynamic states. This system can be applied to various types of membranes composed of polymerized molecules with a broad molecular weight distribution. In low-viscosity media, such as water, the intrinsic motion of gold nanoparticles may become more pronounced, potentially resulting in a more direct and efficient propagation of the object’s motion to the gold nanoparticles. Further investigation is required to assess the significance of each parameter and distinguish meaningful data from less relevant noise.

## 5. Conclusions

This study aimed to develop a novel technique for directly observing nanoparticle motion in real space using TEM, successfully acquiring valuable insights into temperature-dependent membrane dynamics. By combining these observations with DSC measurements, we were able to correlate dynamic quantities with calorimetric data. The fact that these results were obtained using widely available conventional TEM equipment suggests that this method can be widely adopted. This approach is expected to expand its applicability to dynamic measurements of various polymers, offering new perspectives in materials science.

## Data Availability

The original contributions presented in this study are included in the article and Supplementary Material. Further inquiries can be directed to the corresponding author.

## Supplementary Materials

The following supporting information can be downloaded at https://www.mdpi.com/article/10.3390/polym17030292/s1, Figure S1: MSD analysis; Figure S2: Heating and cooling rate of samples; Figure S3: Repeating heating and cooling of PC_8_FA; Figure S4: MSD curves of different-sized nanoparticles during the heating cycle of PC_8_FA; Video S1: 10-s sequence of images from the heating process of PC_8_FA, 20 °C; Video S2: 10-s sequence of images from the heating process of PC_8_FA, 50 °C; Video S3: 10-s sequence of images from the heating process of PC_8_FA, 82.5 °C.
