# Supplementary material for: Real-Time Observation of Polymer Fluctuations During Phase Transition Using Transmission Electron Microscope
Source: Polymers (Basel). 2025 Jan 23;17(3):292. doi: 10.3390/polym17030292 (PMC11820666; doi:10.3390/polym17030292)
Supplement: Supplementary file 1 [file polymers-17-00292-s001.zip › polymers-3404715-Supplementary figures.pdf]

Supplementary figures

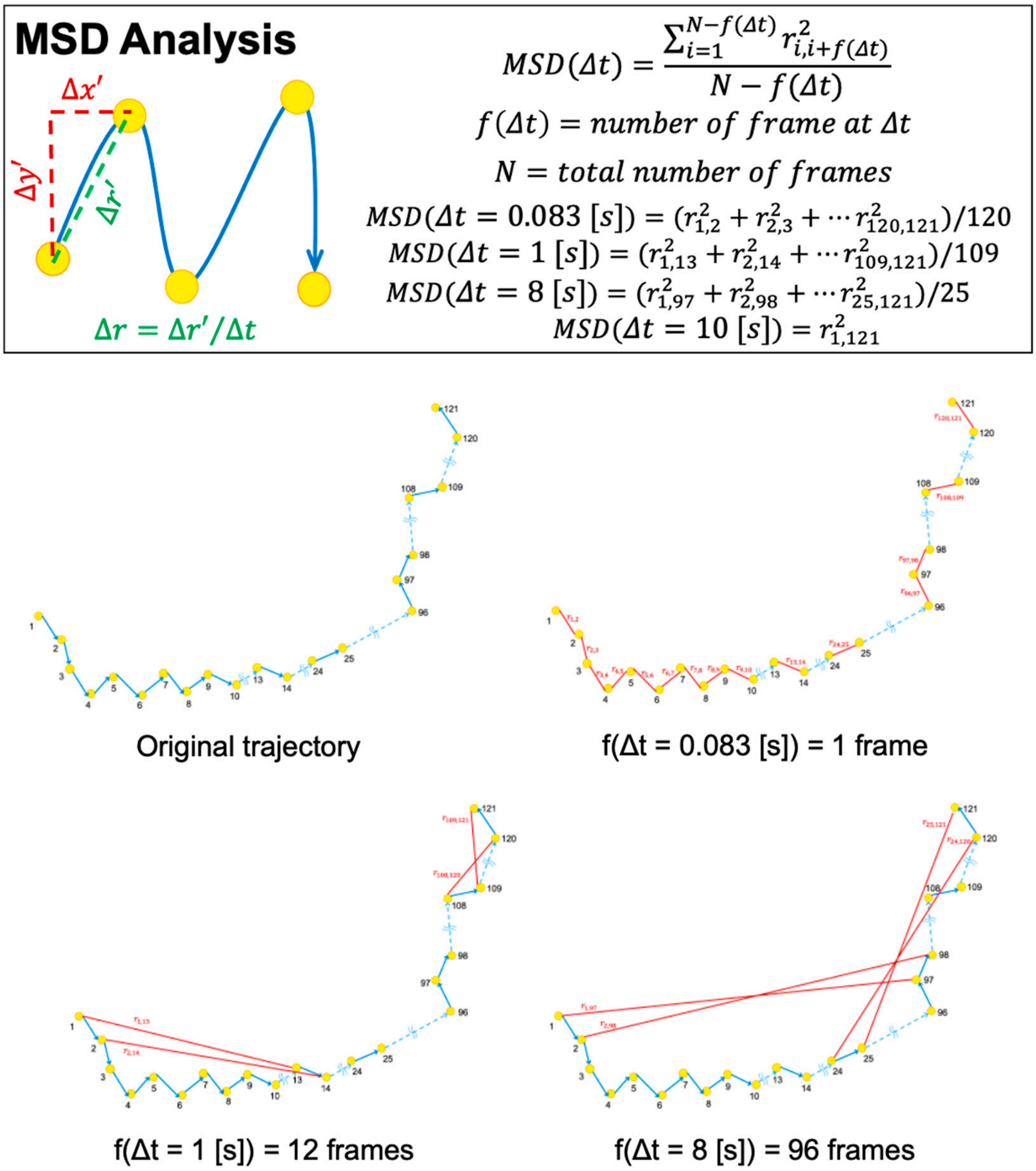

Supplementary Figure S1 | MSD analysis.

Gold nanoparticles were used to observe thermal-induced polymer fluctuation using the electron microscopy. From the video data, MSD of the gold nanoparticle movement was analyzed.

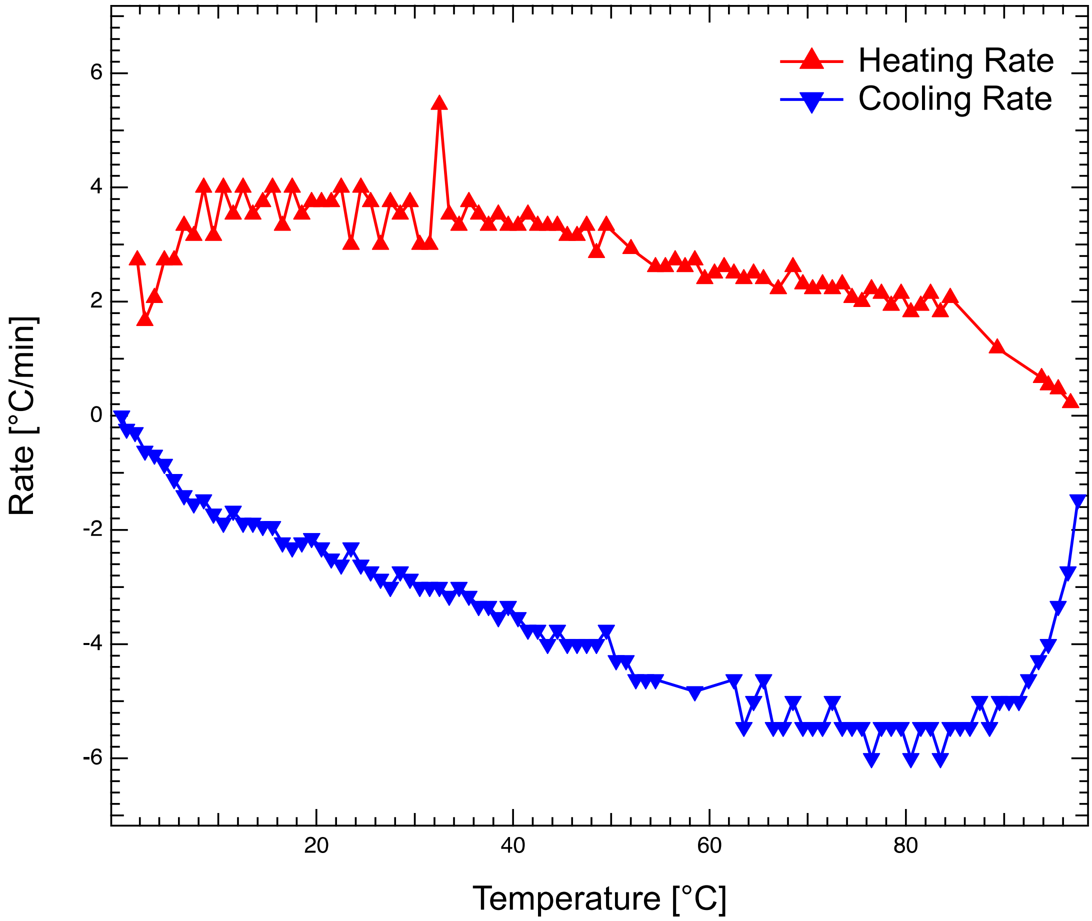

Supplementary Figure S2 | Heating and cooling rate of samples.
The heating rate of the equipment was shown to be 3°C to 5°C/min, while the cooling rate ranged from -2°C to -6°C/min.

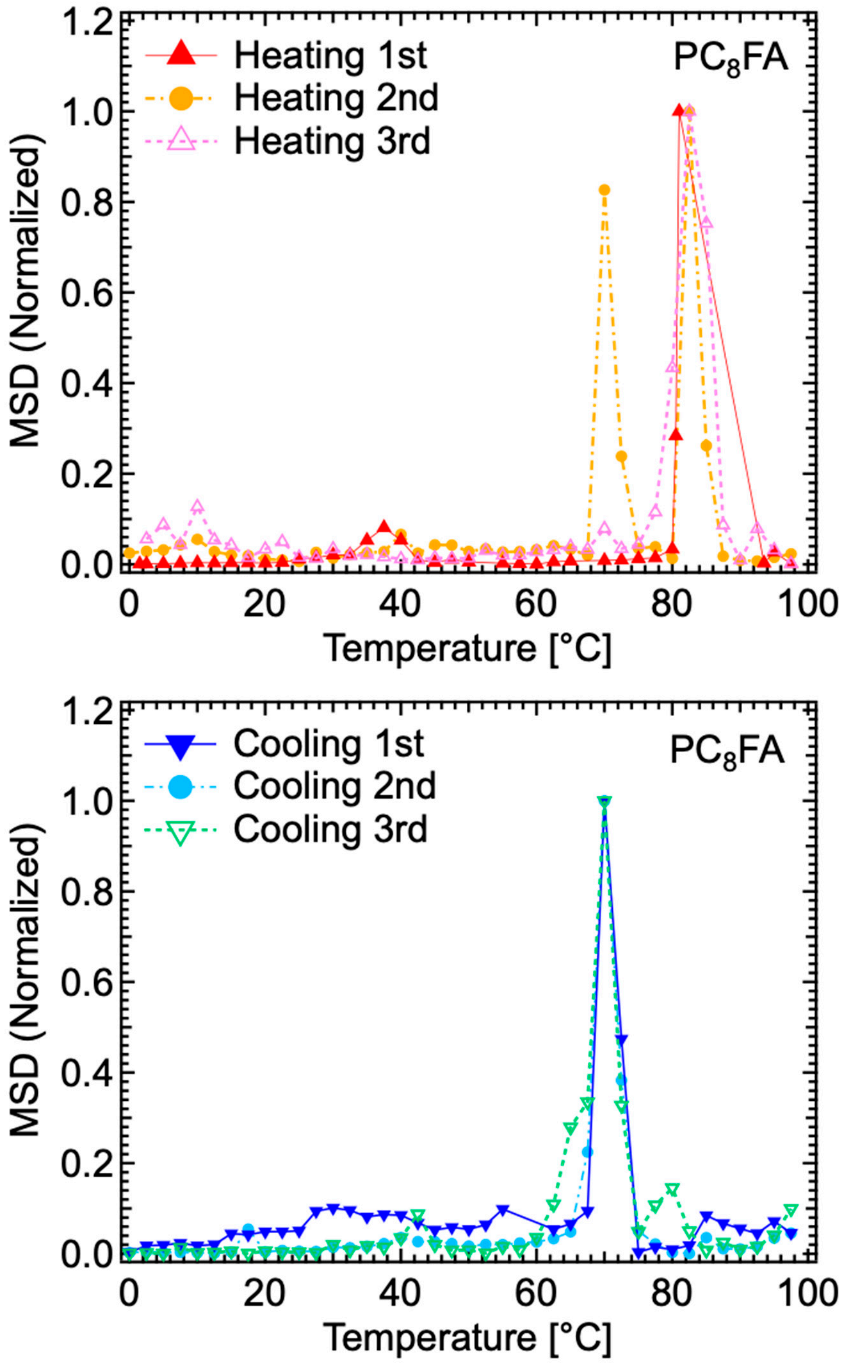

Supplementary Figure S3 | Repeating heating and cooling of PC_8FA. Heating and cooling cycles were repeated several times. As the crystallographic quality of PC_8FA improves through annealing, the dynamics of the third cycles were used for analysis.

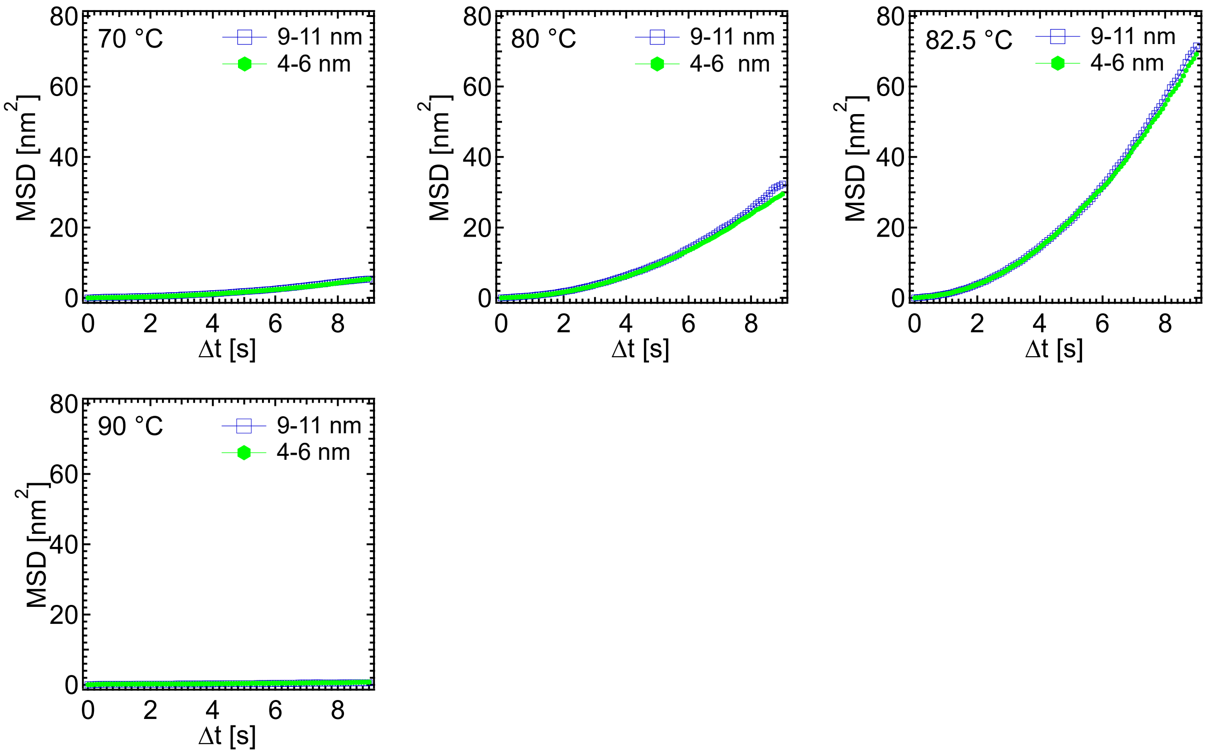

Supplementary Figure S4 | MSD curves of different-sized nanoparticles during the heating cycle of PC$_8$FA.

The MSD curves of small particles (4–6 nm diameter, mostly individual gold nanoparticles) and large particles (9–11 nm diameter, primarily aggregates of 2–3 gold nanoparticles) were compared. No significant differences were observed between them.
